# Completeness, consistency and duplication of records of accidents involving exposure to biological material in dental settings: a descriptive study, Belo Horizonte, Brazil, 2006-2019

**DOI:** 10.1590/S2237-962220v35e20250762.en

**Published:** 2026-05-08

**Authors:** Ana Carolina Marques Medeiros, Victor Santos Batista, Luis Otávio Pereira da Silva, Juliana Vaz de Melo Mambrini, Rafaela Silveira Pinto, Mauro Henrique Nogueira Guimarães Abreu

**Affiliations:** 1 Universidade Federal de Minas Gerais, Belo Horizonte, MG, Brazil Universidade Federal de Minas Gerais Belo Horizonte MG Brazil; 2 Fundação Oswaldo Cruz Instituto René Rachou, Belo Horizonte, MG, Brazil Fundação Oswaldo Cruz Instituto René Rachou Belo Horizonte MG Brazil

**Keywords:** Occupational Exposure, Dentistry, Accidents, Occupational, Data Quality, Descriptive Studies, Exposición Profesional, Odontología, Accidentes de Trabajo, Calidad de los Datos, Estudios Descriptivos.

## Abstract

**Objective:**

To assess duplication, completeness and internal consistency of records of accidents involving exposure to biological material in dental settings in Belo Horizonte from 2006 to 2019.

**Method:**

This is a descriptive study using Notifiable Health Conditions Information System data, including identification, sociodemographic and occupational data, accident circumstances, use of protective equipment, laboratory test, accident response and outcome variables. Duplication was identified by deterministic and probabilistic combinations. Completeness was classified as excellent (≥95%), good (90%-94.9%), fair (70%-89.9%), poor (50%-69.9%), and very poor (<50%). Consistency was evaluated in pairs of variables and classified as excellent (≥90%), fair (70%-89%), and poor (<70%).

**Results:**

1,054 records were investigated, with no duplicates identified. Among the 17 mandatory variables analyzed, 15 showed excellent completeness; the "pregnant" (80.4%) and "issuance of work accident report" (75.5%) variables were classified as fair. Among the essential variables, the fields related to personal protective equipment achieved fair completeness (78.0%-89.9%), while the "injured person laboratory test" and the "source patient" fields, as well as the "response at the time of the accident" and "case evolution" fields, showed very poor completeness (<50%). In terms of consistency, the "source patient known/source patient test results" and the "type of exposure/causative agent" pair were classified as fair, while "case evolution" showed low consistency (55.1%).

**Conclusion:**

Although the system adequately records the occurrence of accidents, weaknesses in clinical and occupational variables reduce its usefulness for surveillance, as they restrict assessment of infection risk and clinical follow-up of exposed workers.

## Resumen

**Objetivo:**

Evaluar la duplicación, la integridad y la consistencia interna de los registros de accidentes con exposición a material biológico en entornos odontológicos de Belo Horizonte, entre 2006 y 2019.

**Método:**

Estudio descriptivo con datos del Sistema de Información de Enfermedades de Declaración Obligatoria, que incluyó variables de identificación, datos sociodemográficos y ocupacionales, circunstancias del accidente, uso de equipos de protección, pruebas de laboratorio, conducta y resultado. La duplicación se identificó mediante combinaciones deterministas y probabilísticas. La integridad se clasificó como excelente (≥95%), buena (90%-94,9%), regular (70%-89,9%), deficiente (50%-69,9%) y muy deficiente (<50%). La consistencia se evaluó en pares de variables y se clasificó como excelente (≥90%), regular (70%-89%) y deficiente (<70%).

**Resultados:**

Se investigaron 1.054 registros, sin identificar duplicados. De las 17 variables obligatorias analizadas, 15 mostraron una completitud excelente; "embarazo" (80,4%) y "emisión del informe de accidente de trabajo" (75,5%) se clasificaron como regulares. Entre las variables esenciales, los campos relacionados con el equipo de protección individual alcanzaron una completitud regular (78,0%-89,9%), mientras que los campos "pruebas de laboratorio del accidentado" y "pruebas de laboratorio del paciente fuente", así como "conducta en el momento del accidente" y "evolución del caso", mostraron una completitud muy baja (<50%). En cuanto a la consistencia, los pares "paciente fuente conocido/resultados de prueba del paciente fuente" y "tipo de exposición/agente causal" se clasificaron como regulares, y "evolución del caso" mostró una consistencia baja (55,1%).

**Conclusión:**

Si bien el sistema registra adecuadamente la ocurrencia de accidentes, las deficiencias en las variables clínicas y ocupacionales reducen su utilidad para la vigilancia, ya que limitan la evaluación del riesgo de infección y el seguimiento clínico de los trabajadores expuestos.

**Ethical aspects**

This research respected ethical principles, having obtained the following approval data:

Research ethics committee: Universidade Federal de Minas Gerais

Opinion number: 6,097,439

Approval date: 2/6/2023

Research ethics committee: Secretaria Municipal de Saúde de Belo Horizonte

Opinion number: 6,161,565

Approval date: 4/7/2023

Certificate of presentation of ethical assessment

68702123.9.0000.5149

68702123.9.3001.5140

Informed consent record: Dismissed.

## Introduction 

Exposure to biological material is a global problem among healthcare professionals, with a cumulative career prevalence of 56.6%^1^. These accidents imply risk of HIV, hepatitis B virus and hepatitis C virus seroconversion, in addition to healthcare costs, absence from work and psychological impacts^2^. In the field of dentistry, this risk is particularly high, given the frequent use of sharp instruments and performance of invasive procedures^3^. Systematic reviews indicate that global prevalence of percutaneous injuries among dental students is 44%^4^, with a similar proportion among assistants, especially in activities such as cleaning instruments and handling anesthesia syringes, considered to be of higher risk^5^. Compared to various categories of healthcare professionals, dentists presented the highest prevalence, totaling 59% of reported cases^3^.

In Brazil, between 2006 and 2022, 36,171 (4.5%) work-related accidents resulting from dental procedures were reported^6^, which highlighted the magnitude of the problem in the country. In Belo Horizonte, Minas Gerais state, between 2019 and 2024, the tabulation platform of the Brazilian Unified Health System Information Technology Department identified 8,607 accidents, of which 366 (4.3%) occurred during dental procedures^7^. These data highlight the relevance of continuous surveillance of this health problem in the context of occupational health.

Given the magnitude of accidents reported in both Brazil and Belo Horizonte, systematic monitoring of cases becomes essential. At the national level, the Notifiable Health Conditions Information System (*Sistema de Informação de Agravos de Notificação*, SINAN) is the main tool for monitoring diseases and guiding surveillance actions^8^^,^^9^. The quality of the information recorded can be assessed based on three fundamental attributes: completeness, internal consistency and duplication^10^^,^^11^. Between 2010 and 2015, analyses of reports of accidents involving exposure to biological material revealed variations in classification of the completeness of essential variables, which ranged from excellent to very poor, without examining consistency or duplication^12^.

Although the quality of health information systems has been gaining prominence in discussions about surveillance, accidents involving exposure to biological material in dental environments require specific analyses that consider record performance in relation to essential quality attributes. Assessing these components of the case reporting system is fundamental for qualifying the use of data in the formulation of health actions and policies. In view of this, this study aimed to assess duplication, completeness and internal consistency of records of accidents involving exposure to biological material in dental environments in Belo Horizonte from 2006 to 2019.

## Methods 

Design

This is a descriptive study. 

This study has been reported as per the REporting of studies Conducted using Observational Routinely-collected health Data (RECORD) Statement^13^.

Setting

We used SINAN data on accidents involving exposure to biological materials in dental environments in Belo Horizonte from 2006 to 2019. This time range corresponds to the availability of the computerized municipal database and precedes the distortions caused by the COVID-19 pandemic^14^. Access to the database took place on August 1, 2023.

The database was provided by the Belo Horizonte City Health Department, without prior exclusion of duplicates, and the final selection was validated by the Occupational Health Coordination.

Participants

The participants corresponded to reported cases of accidents involving exposure to biological material in dental environments in the municipality of Belo Horizonte. Records were included that presented at least one of the following characteristics: occupation, according to the Brazilian Classification of Occupations, namely, dental surgeon (code 2.232), oral health technician, oral health assistant, dental attendant or assistant (code 3.224); the National Classification of Economic Activities in the dental area (codes 8630-5/04, 8650-0/03 and others identified by complementary evidence); the accident circumstances (code 13); or remarks made in the notes fields using text filters ("odont*" or "dent*"). Case reports that did not meet at least one of these four previous characteristics were excluded.

Variables

The variables were classified as mandatory (fields the completion of which is indispensable for validation of the case report) and essential (information relevant for epidemiological analysis, but not preventing data entry), according to the system definitions^8^^,^^9^. The system classification and codes are described in Supplementary Table 1. The variables followed the definitions of the technical manual entitled Accident with Exposure to Biological Material^9^, prepared by the Ceará State Health Department, adopted because it specifically addresses this health problem. The definitions were used in accordance with Ministry of Health manuals^8^^,^^15^, which present conceptual and methodological guidelines aimed at the surveillance of health problems and assessment of the quality of information in health systems.

Data source and measurement 

Data quality was analyzed considering three attributes: duplication^10^^,^^11^, completeness^10^^,^^11^and consistency^10^^,^^11^, as operationalized in national studies^10^^,^^11^. Duplication was defined as records of the same accident reported more than once for the same patient within a short interval^8^. Checking occurred in two stages^8^: deterministic, by direct cross-referencing of name, date of birth, sex and date of accident; and probabilistic, using Soundex and phonetic algorithms to identify similar records, despite spelling variations.

Completeness was assessed by the proportion of valid data in each variable, based on the understanding that incomplete data are blank fields and fields coded as "unknown"^8^. The cut-off points adopted by the Ministry of Health manual were applied^8^, which classifies each variable assessed by the percentage of complete data: ≥95% (excellent); 90%-94.9% (good); 70%-89.9% (fair); 50%-69.9% (poor); <50% (very poor). For the "pregnant" variable, the "not applicable" category, assigned to male records, was considered as valid data^8^.

Internal consistency was defined as coherence between variables that must present a logical relationship^8^ based on technical-epidemiological fundamentals of the Ministry of Health manual on exposure to biological material^15^ and the system's quality manuals^8^^,^^9^. The following combinations were tested: sex and pregnancy; type of exposure and accident circumstances; type of exposure and causative agent; known source patient and laboratory results; age and length of service; outsourcing and name of employing company; case evolution. The operational criteria and examples of incompatibilities are described in Supplementary Table 2. When consistency of the combinations was ≥90%, it was classified as excellent; when it was between 70%-89%, it was considered fair; and when it was <70%, it was classified as low^16^.

Bias control 

A time window of up to seven days between case reports for the same individual was adopted as a complementary criterion for detecting repetitions resulting from typing errors, without confusing distinct events. This time interval was defined based on the understanding that the system manual exemplifies duplications in acute injuries when case reports occur in the same epidemiological week, a temporal unit officially equivalent to seven consecutive days^17^. As accidents with biological material are acute events requiring immediate reporting^17^, the adoption of this time interval is consistent with the nature and operational cycle of the injury. Records outside this period were also checked in deterministic and probabilistic stages, which ensured identification of non-temporal duplications.

Study size

The study population consisted of all reported accidents involving exposure to biological material in dental environments in Belo Horizonte.

Statistical methods

The data were provided in Excel format (Microsoft Corp., Redmond, WA, USA) and processed in Python (version 3.13.1; Python Software Foundation) using the Pandas (2.2.2) and NumPy (1.26.4) libraries. The steps included standardization of categories and codes for unknown values, text standardization, removal of blanks, conversion of dates to an appropriate format, calculation of age at the time of the accident and length of service in completed years. Derived variables were also created, such as the composite key for duplicate detection. When age was missing, it was recalculated from the date of birth and the date of the accident. Impossible values, such as length of service exceeding age, were recoded as invalid. The validation, standardization and cleansing steps of the consolidated database are summarized in Figure 1.

The results were expressed as absolute numbers and percentages with regard to duplication, completeness and consistency. Scripts and documentation of the methodological flow are included in the supplementary material.

## Results 

Of the total 12,717 records of accidents involving exposure to biological material, 1,054 met the study's inclusion criteria and were included in the analysis (Supplementary Figure 2). No duplicates were observed, and two additional records corresponded to distinct accidents involving the same worker.

Table 1 shows the completeness of the mandatory and essential variables. Among the mandatory variables, 15 of the 17 fields showed completeness classified as excellent, while the "pregnant" variable (80.4%) and the "issuance of work accident report" variable (75.5%) were classified as fair. Among the essential variables, fields related to address (street number complement, reference point and postcode) showed completeness ranging from poor to very poor, while "direct distance dialing code" was classified as fair. Contact variables, such as telephone number and area of residence, were classified as fair. Regarding the 12 work-related variables, there was a predominance of poor (41.6%) and fair (33.3%) classifications.

Table 2 summarizes the essential exposure variables: accident characterization, use of personal protective equipment (PPE), and laboratory tests. A good proportion of records were observed for percutaneous injuries (90.8%), a poor proportion for mucosal exposure (52.4%), and a very poor proportion for non-intact skin (49.7%). Variables related to PPE use, including gloves, gowns, goggles and masks, as well as "known source patient", showed fair completeness (78.0%-89.9%). The remaining clinical variables showed the lowest completion rates and all were classified as having very poor completeness (<50%).

Table 3 shows the results of the consistency assessment. Most combinations were classified as excellent, while "known source patient and laboratory results" (13.1%) and "type of exposure and causative agent" (13.5%) showed fair consistency. The "case evolution" variable showed low consistency (55.1%).

**Table 1:** Completeness of identification, demographic, residence and work variables in records of accidents involving exposure to biological material in dental environments. Belo Horizonte, 2006-2019 (n=1,054)

Variable	n (%)	Classification^a^
Obligator		
Event report identification variables		
Report number	1,054 (100.0)	Excellent
Condition/disease name	1,054 (100.0)	Excellent
Report date	1,054 (100.0)	Excellent
Federative Unit abbreviation	1,054 (100.0)	Excellent
Reporting unit municipality	1,054 (100.0)	Excellent
Reporting unit National Health Establishment Registry	1,054 (100.0)	Excellent
Date of accident	1,054 (100.0)	Excellent
Demographic and residence variables		
Patient's full name	1,054 (100.0)	Excellent
Date of birth	1,054 (100.0)	Excellent
Age (years, whether date of birth unknown)	1,054 (100.0)	Excellent
Patient's sex	1,054 (100.0)	Excellent
Pregnant	847 (80.4)	Fair
Patient's Federative Unit of residence	1,054 (100.0)	Excellent
Municipality of residence code	1,054 (100.0)	Excellent
Country of residence (if foreign)	1,054 (100.0)	Excellent
Occupation identification	1,054 (100.0)	Excellent
Issuance of work-related accident report	796 (75.5)	Fair
Essential		
Race/skin color	909 (86.2)	Fair
Level of schooling	1,040 (98.7)	Excellent
Mother's full name	1,054 (100.0)	Excellent
Name of district of residence	854 (81.0)	Fair
Name of neighborhood	1,047 (99.2)	Excellent
Residence street type	1,046 (99.1)	Excellent
Street number	1,039 (98.6)	Excellent
Street number complement	556 (52.8)	Poor
Residence reference point	274 (26.0)	Very poor
Postcode	649 (61.6)	Poor
Direct distance dialing code	868 (82.3)	Fair
Patient's telephone number	866 (82.2)	Fair
Patient's area of residence	881 (83.5)	Fair
Work variables		
Employment situation	985 (93.5)	Good
Length of time in occupation (years)	1,039 (98.6)	Excellent
Identification of employing company	628 (59.6)	Poor
Corporate name of employing company	1,054 (100.0)	Excellent
National classification of economic activities	757 (71.8)	Fair
Federative Unit where company is located	844 (80.1)	Fair
Employer municipality	808 (76.7)	Fair
District in municipality	689 (65.4)	Poor
Neighborhood in municipality	691 (65.6)	Poor
Company address	694 (65.8)	Poor
Number of company premises	689 (65.4)	Poor
Outsourced company	893 (84.7)	Fair

a Classification of completeness: Excellent ≥95%; Good 90%-94.9%; Fair 70%-89.9%; Poor 50%-69.9%; Very poor <50%.

**Table 2:** Completeness of exposure variables, accident characterization and clinical outcomes in accidents involving biological material in dental environments. Belo Horizonte, 2006-2019 (n=1,054)

Variable	n (%)	Classification^a^
Essential		
Type of exposure		
Percutaneous	957 (90.8)	Good
Mucous membrane	552 (52.4)	Poor
Intact skin	640 (60.7)	Poor
Non-intact skin	524 (49.7)	Very poor
Other	347 (32.9)	Very poor
Accident characterization		
Type of organic material	954 (90.5)	Good
Accident circumstances	1,038 (98.5)	Excellent
Accident causative agent	997 (94.6)	Good
Use of personal protective equipment		
Gloves	947 (89.9)	Fair
Gown	840 (79.7)	Fair
Goggles	822 (78.0)	Fair
Mask	836 (79.3)	Fair
Facial protection	719 (68.2)	Poor
Boots	695 (65.9)	Poor
Laboratory tests/vaccination status		
Hepatitis B vaccination status	873 (82.8)	Fair
Test results of the injured person (Date zero)		
Human immunodeficiency virus antibody detection test	517 (49.0)	Very poor
Hepatitis B virus surface antigen	484 (45.9)	Very poor
Hepatitis B virus surface antigen antibody detection test	437 (41.4)	Very poor
Hepatitis C virus antibody detection test	483 (45.8)	Very poor
Source patient known	922 (87.4)	Fair
Source patient test results		
Hepatitis B virus surface antigen	394 (37.4)	Very poor
Human immunodeficiency virus antibody detection test	454 (43.0)	Very poor
Hepatitis B core antigen antibody detection test	326 (30.9)	Very poor
Hepatitis C virus antibody detection test	368 (34.9)	Very poor
Response at the time of the accident		
Chemoprophylaxis not indicated	449 (42.6)	Very poor
Indicated chemoprophylaxis refused	103 (9.8)	Very poor
Zidovudine and lamivudine	199 (18.9)	Very poor
Zidovudine, lamivudine and indinavir	133 (12.6)	Very poor
Zidovudine, lamivudine and nelfinavir	107 (10.1)	Very poor
Hepatitis B immune globulin	107 (10.1)	Very poor
Hepatitis B vaccine	108 (10.2)	Very poor
Other antiretroviral regimen	335 (31.8)	Very poor
Other regimen specification	155 (14.7)	Very poor
Case evolution	473 (44.9)	Very poor

a Classification of completeness: Excellent ≥95%; Good 90%-94.9%; Fair 70%-89.9%; Poor 50%-69.9%; Very poor <50%.

**Table 3:** Inconsistencies between variables in records of accidents involving exposure to biological material in dental environments. Belo Horizonte, 2006-2019 (n=1.054)

Consistency criterion	n^a^ (%^a,b^)	Classification^c^
Sex and pregnancy	59 (5.6)	Excellent
Age (years) and length of time in occupation (years)	94 (9.0)	Excellent
Source patient known and source patient test results	138 (13.1)	Fair
Type of exposure and causative agent	147 (13.5)	Fair
Type of exposure and accident circumstances	38 (3.6)	Excellent
Outsourcing and name of employing company	8 (0.8)	Excellent
Case evolution	581 (55.1)	Low

a Refers to inconsistent records; ^b^Percentages calculated based on records with both variables filled in; ^c^Classification of consistency: Excellent ≥90% of records consistent; Fair 70%-89%; Low consistency <70%.

## Discussion 

Our assessment of records of accidents involving exposure to biological material in dental environments in Belo Horizonte from 2006 to 2019 showed absence of duplicates and excellent completeness in most mandatory variables. With regard to essential variables, variability was observed in the SINAN data, ranging from very poor to excellent. Variables important for the event, such as accident characterization and clinical outcomes of accidents involving biological material in dental environments, frequently showed very poor and poor performance. Internal consistency was classified as fair between "known source patient and laboratory results" and "type of exposure and causative agent"; while "case evolution" showed low coherence. These findings are of concern regarding patient follow-up and epidemiological surveillance of injured oral health professionals.

It is important to note that the results of this study should be interpreted considering limitations inherent to its descriptive design and use of secondary data, which are subject to classification bias, incompleteness and underreporting, especially in recurring or informally reported accidents. Despite these limitations, the findings offered consistent support for improving the case reporting process, strengthening surveillance and guiding prevention strategies. Regarding duplication, the absence of repeated records suggested adequate control of the case reporting flow and effective data consolidation, these being essential components for surveillance systems^18^. Similar findings were described for leprosy in João Pessoa, Paraíba state, between 2001 and 2019, where duplications accounted for 0.3%^10^, and in reported cases of sexual violence against children and adolescents in Santa Catarina state, from 2009 to 2019, in which no duplicate records were identified either^18^, which highlighted the effectiveness of the data cleansing routines. These results highlight the importance of continuously improving duplication procedures and active surveillance.

The identification of two distinct accidents for the same worker reaffirmed the need to distinguish undue repetitions from real recurrences, which indicate persistent occupational risk. This distinction depends on judiciously applied linkage and time checking rules^19^. Short time windows may not capture all repetitions, which highlighted the importance of audits and periodic reinputting to the system^8^^,^^9^^,^^20^.

The completeness of the mandatory variables was mostly excellent, this being a result similar to that observed nationally between 2010-2015 for identification variables in accidents involving exposure to biological material^12^. These results indicated that mandatory fields favor completion, although they do not ensure the quality or accuracy of the records, which depend on the understanding and commitment of the case reporters^8^^,^^12^^,^^21^. The "pregnant" variable and the "issuance of work accident report" variable showed fair completeness, which emphasized that although the fields are mandatory, this does not ensure this information being recorded. At the national level, from 2010 to 2015, the completeness of the "issuance of work accident report" field was <70%^12^, and in a university hospital in Pernambuco state, from 2009 to 2019, 98% of the forms did not contain this information, which made it impossible to analyze legal and labor aspects^22^.

These results reflected lack of integration between surveillance and the labor sector, which made it difficult to trace accidents^22^. The "pregnant" variable also proved critical, as the national protocol provides for specific monitoring and prophylactic adjustments^15^. In dental environments, where women predominate among those exposed and percutaneous accidents are frequent during instrument cleaning and anesthetic syringe handling, the absence of data restricted surveillance of specific risks^5^^,^^23^.

Among the essential variables, injured person laboratory tests, source patient laboratory tests, as well as accident response and evolution showed poor and very poor completeness, this being a pattern similar to the national one^12^. These shortcomings in data completion suggested difficulties in the articulation between surveillance and care, since clinical follow-up is not always recorded as part of the case reporting process^8^^,^^22^.

Type of exposure, a variable determinant of infection risk^15^^,^^20^, showed good completeness for percutaneous accidents, but poor or very poor performance for mucosal and cutaneous exposures. This pattern repeated national findings^12^ and international reviews on dentistry^3^^,^^23^. As it involves higher risk of transmission of HIV, HBV and HCV viruses, this type of accident tends to be more readily reported, which may reflect a greater perception of risk among professionals^12^^,^^22^^,^^23^.

Absence of a high percentage of laboratory result data and case evolution data, such as found in the study, can also compromise surveillance and make it impossible to monitor seroconversion, which is essential for characterizing the real risk of infection^12^^,^^15^^,^^22^. This gap may result from recording failures, when tests and accident responses are performed but not reported, or from the effective absence of testing and follow-up. In the first case, the statistics lose reliability^8^^,^^12^^,^^21^; in the second case, there is failure in care, in disagreement with protocols that provide for testing the source and injured person and follow-up for up to six months^15^^,^^20^. International reviews have associated low completeness with the loss of secondary utility of data, especially in clinical fields^24^, and have reinforced the need for more functional control routines and interfaces^24^^,^^25^.

The "accident response" variable, which records the measures taken after the accident, is essential for evaluating post-exposure management^9^^,^^15^. A recent review reported that about one third of sources are not tested for HIV, HBV or HCV viruses, usually due to refusal or lack of consent^26^. Studies have also pointed to underreporting among students and assistants, which hinders post-exposure assessments^1^^,^^23^. This pattern suggests persistent failures in biosafety culture, even in scenarios with consolidated guidelines^15^^,^^21^.

Assessment of "case evolution" is also critical, as it is responsible for indicating whether there was follow-up or dropout and a high proportion of blank records. This suggests recording failures or lack of follow-up, in disagreement with national and international protocols^9^^,^^15^^,^^21^, which compromises assessment of the effectiveness of the prophylaxis indicated^21^^,^^22^.

Regarding occupational variables, most ranged in completeness from fair to poor, which makes it difficult to identify higher-risk environments and limits the planning of preventive measures^27^. This limitation is relevant because working conditions and adherence to standards influence the occurrence and recording of exposures^1^. Lack of information on personal protective equipment also restricts analyses of adherence to protective barriers^15^^,^^23^. An international study demonstrated that variations in completeness resulted from lack of standardization, conceptual gaps and insufficient training of reporting professionals^28^.

Regarding consistency, "case evolution" was classified as low, which may compromise its function of synthesizing outcomes and the usefulness of the system for assessing the effectiveness of prophylactic measures^15^^,^^20^. The consistency of the "known source patient and laboratory results" and "type of exposure and causative agent" pairs was classified as fair and may also affect assessment of biological risk and definition of post-exposure accident responses^15,20^, in addition to hindering the targeting of preventive practices^8^^,^^9^. Consistency is considered one of the most influential dimensions of data quality, as it affects completeness, accuracy, timeliness and accessibility^24^. Inconsistencies in data capture and lack of standardization compromise coherence between fields, as observed in the pairs assessed^24^.

Understanding the scenario of weaknesses in the completeness and internal consistency of essential variables in dental environments is fundamental for identifying system limitations and guiding more effective surveillance strategies^26^. The weaknesses observed indicated misalignment between regulations and daily practices, which stressed the need for continuous improvement of record-keeping^8^^,^^21^. Surveillance can be strengthened by expanding active actions, which ensures adequate follow-up and outcome monitoring^22^. International experience has shown that quality improvement depends on interventions at all stages of the process, such as checking automation, field standardization and periodic training^28^.

Continuous team training, form simplification and checking routine automation are suggested, which increase the usefulness of information for accident prevention, especially in higher risk scenarios. Periodic system evaluations remain essential for monitoring progress and guiding continuous adjustments^8^^,^^21^.

In Belo Horizonte, Minas Gerais, the SINAN records adequately recorded the occurrence of accidents involving exposure to biological material in dental environments, as evidenced by the absence of duplicates and identification of actual recurrences. However, weaknesses persisted in clinical and occupational variables, reducing the system's applicability for health surveillance. Insufficient completion and inconsistency in fields such as "laboratory tests", "accident response" and "case evolution" indicated structural limitations in the case reporting process, compromising assessment of real risk of infection and clinical follow-up of exposed workers.

## Data Availability

The data analyzed are administrative and confidential, originating from the Notifiable Health Conditions Information System under the management of the Belo Horizonte City Health Department. Direct access to the data depends on authorization from the competent authority. The analysis procedures, including files generated in the checking and processing stages, are described in this manuscript and in the supplementary material. Requests for access may be assessed by the authors, according to legal confidentiality criteria and editorial review.
