# Supplementary material for: Completeness, consistency and duplication of records of accidents involving exposure to biological material in dental settings: a descriptive study, Belo Horizonte, Brazil, 2006-2019
Source: Epidemiol Serv Saude. 2026 May 8;35:e20250762. doi: 10.1590/S2237-962220v35e20250762.en (PMC13686414; doi:10.1590/S2237-962220v35e20250762.en)
Supplement: Supplementary file 1 [file 2237-9622-ress-35-e2025762-Figuras-Suplementares-EN.pdf]

Supplementary Figure 1. Validation and preparation stages for the consolidated database used for duplication, completeness and consistency analysis. Belo Horizonte, 2006-2019 (n=1,054)

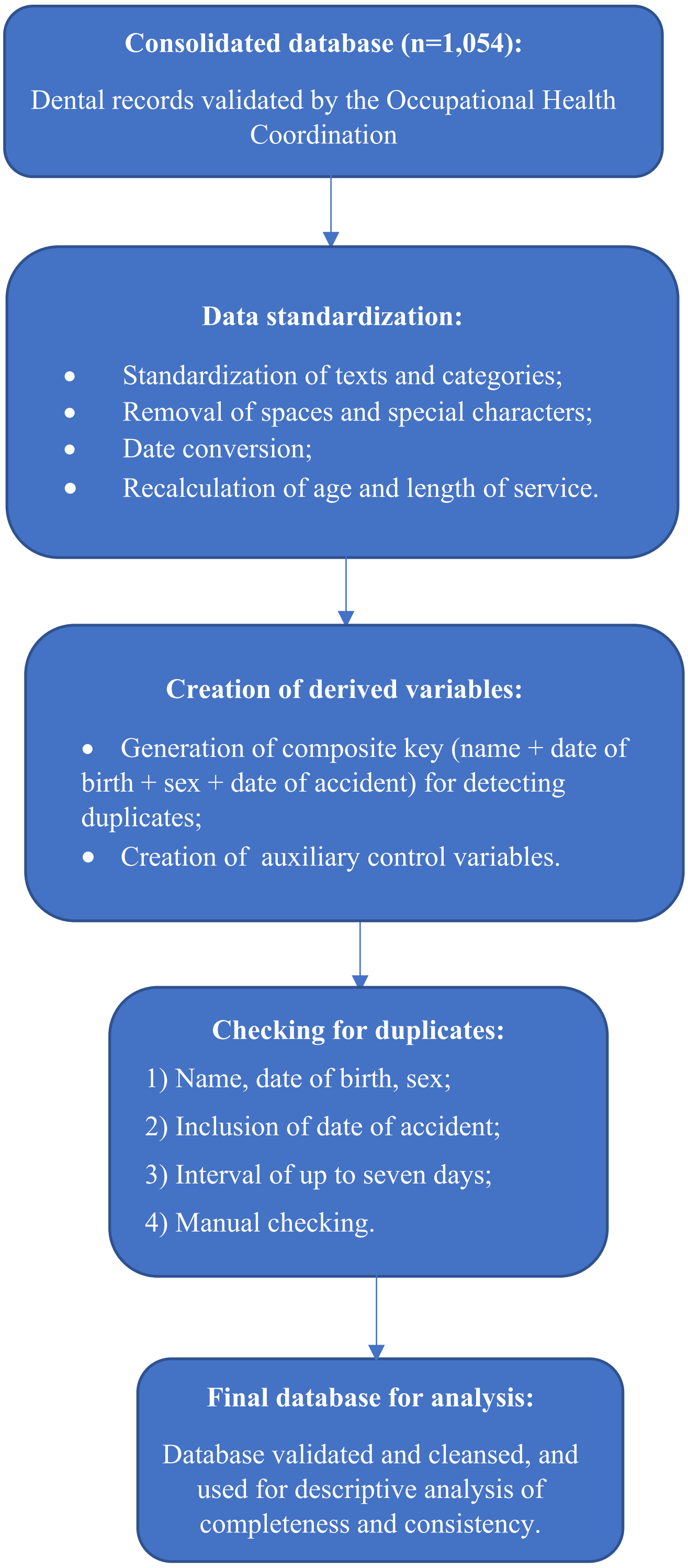

Supplementary Figure 2. Criteria for inclusion, exclusion and filtering of records of accidents involving exposure to biological material in dental environments. Belo Horizonte, 2006-2019 (n=12,717)

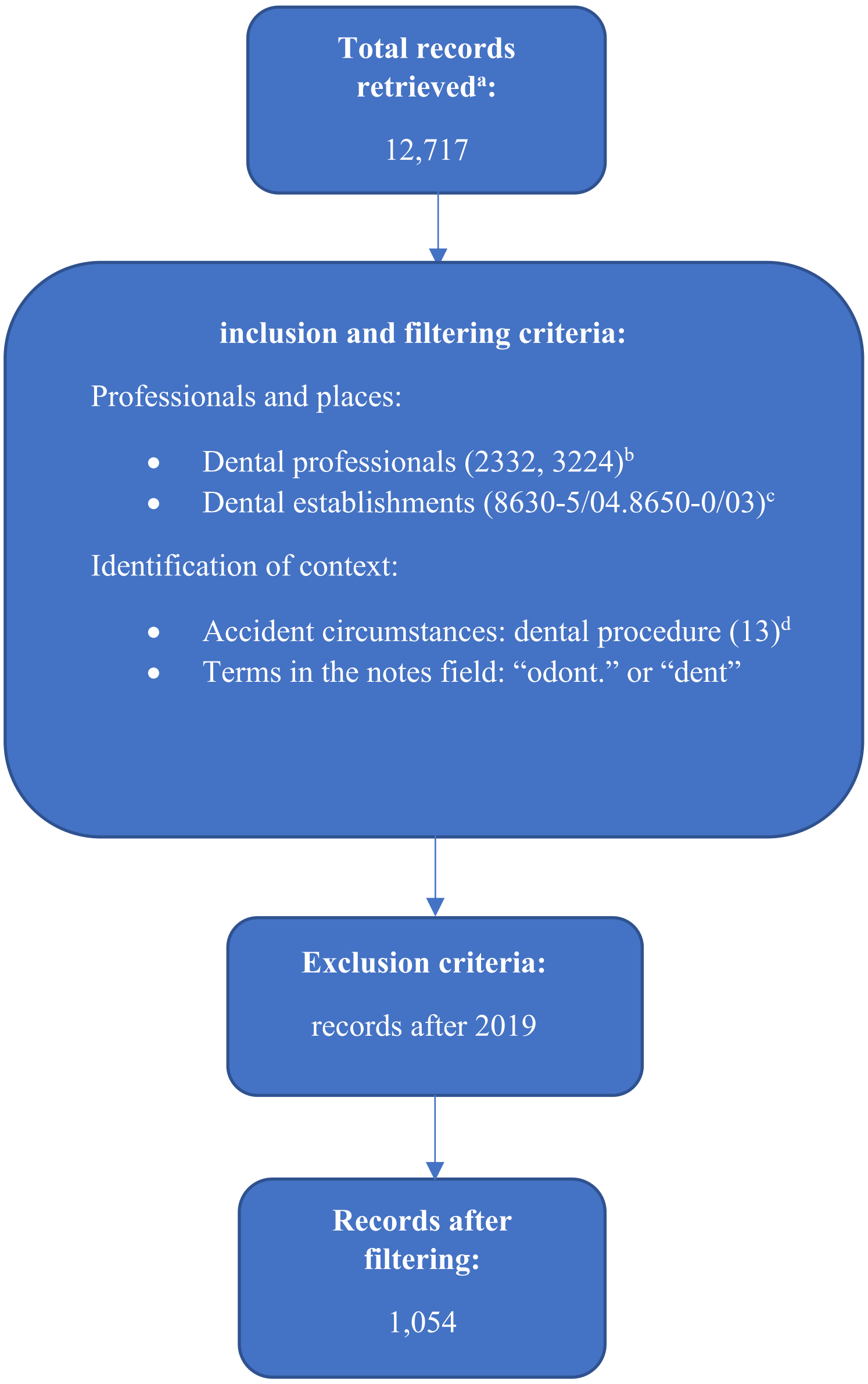

Note: [a]Total records retrieved corresponds to the crude database provided by the Belo Horizonte City Health Department, without prior exclusion of duplicates or filtering by occupation; [b]Brazilian Occupation Classification Codes; [c]National Economic Activity Classification Codes; [d]Code on the case report form.
