# Supplementary material for: Completeness, consistency and duplication of records of accidents involving exposure to biological material in dental settings: a descriptive study, Belo Horizonte, Brazil, 2006-2019
Source: Epidemiol Serv Saude. 2026 May 8;35:e20250762. doi: 10.1590/S2237-962220v35e20250762.en (PMC13686414; doi:10.1590/S2237-962220v35e20250762.en)
Supplement: Supplementary file 2 [file 2237-9622-ress-35-e2025762-Figuras-Suplementares-PT.pdf]

Figura suplementar 1. Etapas de validação e de preparo do banco consolidado utilizado para análise de duplicidade, completitude e consistência. Belo Horizonte, 2006-2019 (n=1.054)

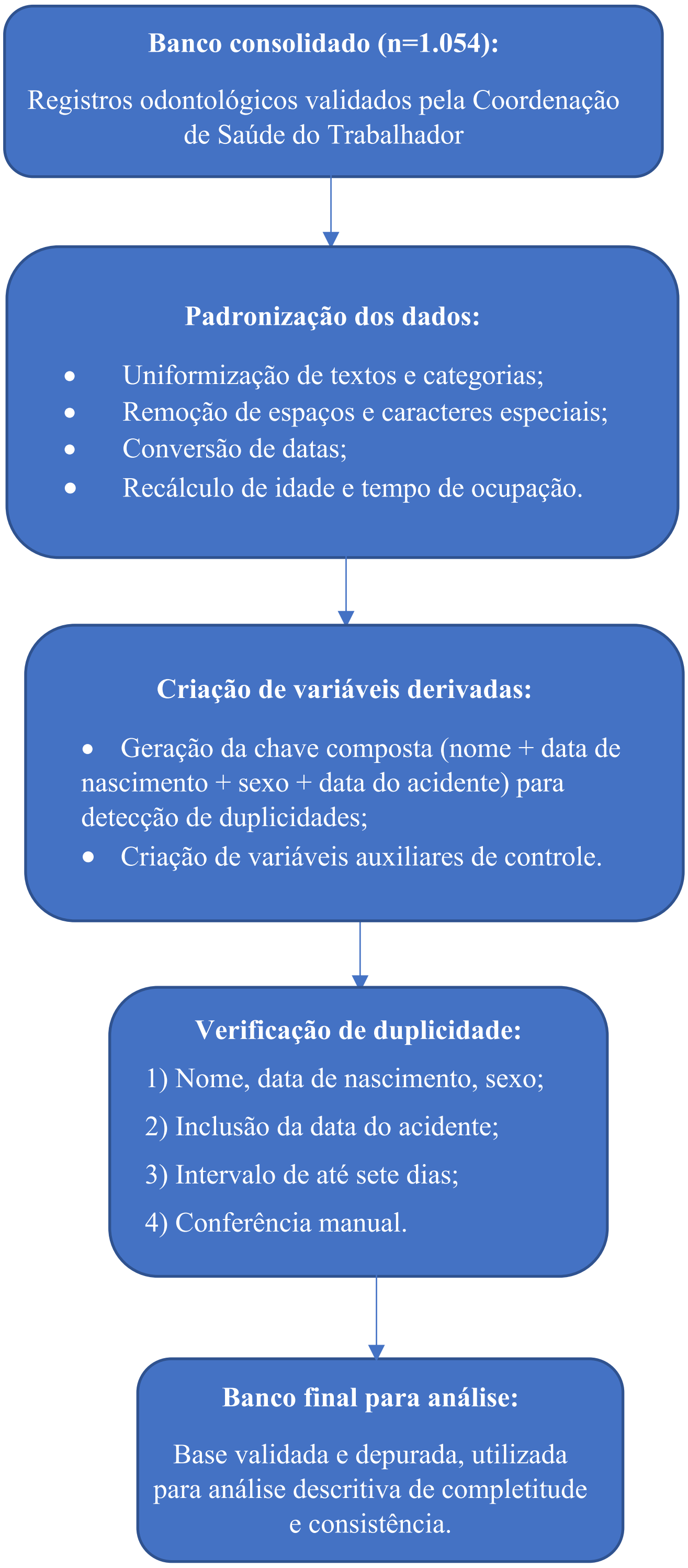

Figura suplementar 2. Critérios de inclusão, exclusão e filtragem de registros de acidentes com exposição a material biológico em ambientes odontológicos. Belo Horizonte, 2006-2019 (n=12.717)

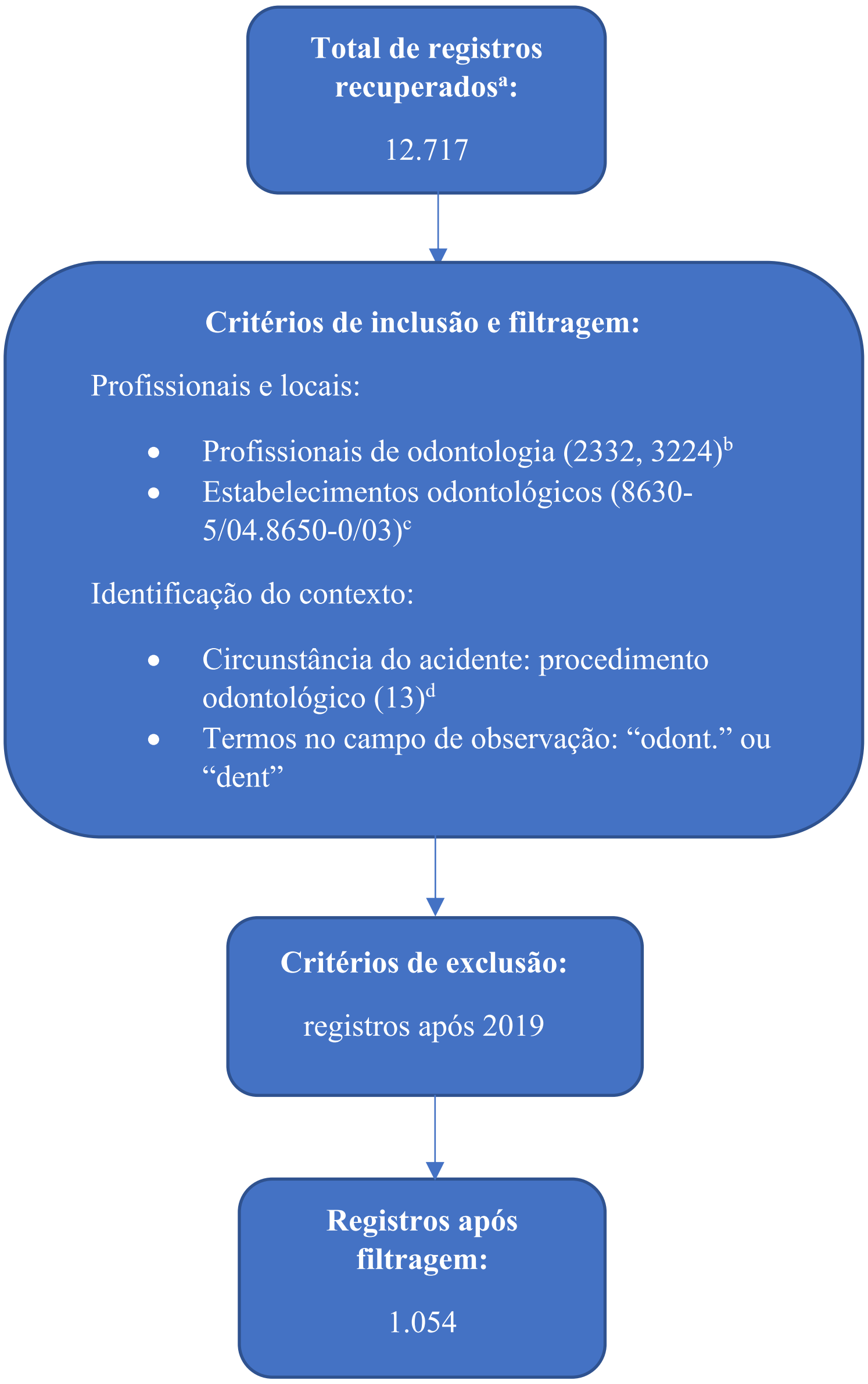

Nota: [a]Total de registros recuperados corresponde ao banco bruto fornecido pela Secretaria Municipal de Saúde de Belo Horizonte, sem exclusão prévia de duplicidades ou filtros por ocupação; [b]Códigos da Classificação Brasileira de Ocupações; [c]Códigos da Classificação Nacional de Atividades Econômicas; [d]Código na ficha de notificação.
