# Supplementary material for: Completeness, consistency and duplication of records of accidents involving exposure to biological material in dental settings: a descriptive study, Belo Horizonte, Brazil, 2006-2019
Source: Epidemiol Serv Saude. 2026 May 8;35:e20250762. doi: 10.1590/S2237-962220v35e20250762.en (PMC13686414; doi:10.1590/S2237-962220v35e20250762.en)
Supplement: Supplementary file 3 [file 2237-9622-ress-35-e2025762-Tabelas-Suplementares-EN.pdf]

**Supplementary Table 1.** Variables analyzed and respective categories in records of accidents involving exposure to biological material in dental environments. Belo Horizonte, 2006-2019

| Variable name | Classification | Categories/values (codes)[a] |
|---|---|---|
| **Event report identification variables:** | | |
| Report number | Mandatory | Open field (system identifier) |
| Condition/disease name | Mandatory | Occupational accident with exposure to biological material (fixed text) |
| Report date | Mandatory | Date (DD/MM/YYYY) |
| Federative Unit abbreviation[b] | Mandatory | Abbreviation and/or Brazilian Institute of Geography and Statistics code |
| Reporting unit municipality[b] | Mandatory | Municipality name and Brazilian Institute of Geography and Statistics code |
| Reporting unit national health establishment registration number[b] | Mandatory | Specific code |
| Date of accident | Mandatory | Date (DD/MM/YYYY) |
| **Demographic and residence variables:** | | |
| Patient's full name | Mandatory | Open field |
| Date of birth | Mandatory | Date (DD/MM/YYYY) |
| Age (if date of birth unknown) | Mandatory | Number (years) |
| Patient's sex | Mandatory | Male (M); Female (F); Unknown (I) |
| Pregnant | Mandatory | 1-6; 9 (description as per 2019 SINAN form) |
| Patient's Federative Unit (FU) of residence[b] | Mandatory | FU (abbreviation) and/or Brazilian Institute of Geography and Statistics code |
| Municipality of residence code | Mandatory | Brazilian Institute of Geography and Statistics municipality code |
| Country of residence (if foreign)[b] | Mandatory | Open field (country name) |
| Occupation identification | Mandatory | Open field (description); if available, code CBO-2002 |
| Issuance of work-related accident report | Mandatory | 1=Yes; 2=No; 3=Not applicable; 9=Unknown |
| Race/skin color | Essential | 1=White; 2=Black; 3=Asian; 4=Mixed-race; 5=Indigenous; 9=Unknown |
| Level of schooling | Essential | 0-10 (description as per 2019 SINAN form) |
| Mother's full name; name of district of residence; name of neighborhood; street type; street number; street number complement; point of reference | Essential | Open fields |
| Postcode (CEP) | Essential | CEP (numeric) |
| Direct distance dialing code (DDD) | Essential | DDD (numeric) |
| Patient's telephone number | Essential | Telephone number (numeric) |
| Patient's area of residence | Essential | 1=Urban; 2=Rural; 3=Peri-urban; 9=Unknown |
| **Work variables:** | | |
| Employment situation | Essential | 01-12 (description as per 2019 SINAN form) |
| Length of time in occupation | Essential | Number of years (open field) |
| Identification of employing company | Essential | CNPJ/CPF – tax identification number (open field) |
| Corporate name of employing company[b] | Essential | Open field |
| National classification of economic activities[b] | Essential | Specific code |

| | | |
|---|---|---|
| Federative Unit where company is located[b] | Essential | Abbreviation and/or Brazilian Institute of Geography and Statistics code |
| Employer municipality[b] | Essential | Municipality name and Brazilian Institute of Geography and Statistics code |
| District in municipality[b]/Neighborhood in municipality[b]/Company address[b]/Number of company premises[b]/ | Essential | Open fields |
| Outsourced company? | Essential | 1=Yes; 2=No; 3=Not applicable; 9=Unknown |
| **Type of exposure[c]:** | | |
| Percutaneous/Mucous membrane/Intact skin/Non-intact skin/Other | Essential | For each subitem: 1=Yes; 2=No; 9=Unknown |
| **Accident characterization:** | | |
| Type of organic material | Essential | 1-8; 9 (description as per 2019 SINAN form) |
| Accident circumstances | Essential | Code as per 2019 SINAN form: 01-16, 98=Outros, 99=Unknown. (descriptions on the official instrument) |
| Accident causative agent | Essential | 1=Needlestick with lumen; 2=Needlestick without lumen/solid; 3=Intracath; 4=Glass; 5=Blade/lancet; 6=Other; 9=Unknown |
| **Use of personal protective equipment:** | | |
| Gloves/Gown/Goggles/Mask/Facial protection/Boots | Essential | For each subitem: 1=Yes; 2=No; 9=Unknown |
| **Laboratory tests/Vaccination status:** | | |
| Hepatitis B vaccination status | Essential | 1=Vaccinated; 2=Not vaccinated; 9=Unknown (3-dose regimen) |
| **Test results (injured person and source patient) (Date zero):** | | |
| Serology panel (Anti-HIV, HBsAg, Anti-HBs, Anti-HCV) | Essential | For each subitem: 1=Positive; 2=Negative; 3=Inconclusive; 4=Not performed; 9=Unknown |
| Source patient known | Essential | 1=Yes; 2=No; 9=Unknown |
| **Response at time of accident[c]:** | | |
| Multiple items (dichotomous variables): chemoprophylaxis/ARV/regimens/vaccine/IGHB | Essential | For each subitem: 1=Yes; 2=No; 9=Unknown |
| **Case evolution** | Essential | 1-6; 9 (description as per 2019 SINAN form) |
| **Death (if applicable)** | Essential | Captured via 'Case evolution' (5 or 6); 'Date of death' is a date field (DD/MM/YYYY) |

Note: [a]Categories and codes extracted from the "Occupational accident involving exposure to biological material" investigation form held on the Notifiable Health Conditions Information System; [b]Fields without official coding indicated as "open field" or as "external identifier", using the following systems: Brazilian Institute of Geography and Statistics, National Health Establishment Registry, National Classification of Economic Activities and Brazilian Occupation Classification; [In] groups comprised of multiple items, each subitem is recorded as an independent dichotomous variable. Brasil. Ministério da Saúde. Secretaria de Vigilância em Saúde. Ficha de investigação: Acidente de trabalho com exposição a material biológico [Internet]. Brasília: Ministério da Saúde; 2019 Jun 21 [cited 2025 Sep 25]. Available from: https://portalsinan.saude.gov.br/images/DRT/DRT_Acidente_Trabalho_Biologico.pdf.

**Supplementary Table 2.** Criteria for consistency between variables in records of accidents involving exposure to biological material in dental environments. Belo Horizonte, 2006-2019

| Pair of variables | Description of expected relationship | Consistence criterion | Inconsistency criterion |
|---|---|---|---|
| Patient's sex vs. Pregnancy | Consistency between sex and answer to the Pregnancy variable | Records with male sex and Pregnancy variable filled in as "Not applicable" (code 6) | Records with female sex and Pregnancy variable filled in with an incompatible code ("Not applicable", code 6) |
| Type of exposure vs. Accident circumstances | Consistency between type of exposure and informed accident circumstances | For each type of exposure recorded (Mucous membrane, Intact skin, Non-intact skin or Percutaneous), accident circumstances compatible with the system's standardized codes | Type of exposure incompatible with accident circumstances, or records classified as "Unknown" (codes 9 or 99) in an inconsistent manner |
| Source patient known vs. Source patient laboratory tests | Consistency between source patient identification and laboratory test performance | For records with source patient identified as "Yes" (code 1), at least one laboratory test informed with valid value | For records with source patient identified as "Yes" (code 1), all laboratory tests left blank, marked as "Not performed" (code 4) or "Unknown" (code 9) |
| Patient's age (years) vs. Length of service (years) | Consistency between patient's age and recorded length of service | Informed length of service equal to or less than patient's current age and non-negative value | Length of service greater than patient's current age or negative value |
| Outsourced vs. Name of employing company | Consistency between information on outsourcing and answer to the Employing company name field | For records with Outsourcing informed as "Yes" (code 1), Name of employing company field filled in; when Outsourcing ="Unknown" (code 9), Name of employing company field left blank | For records where Outsourcing="Yes" (code 1), Name of employing company field left blank; or where Outsourcing="Unknown" (code 9), Name of employing company filled in |

| Pair of variables | Description of expected relationship | Consistence criterion | Inconsistency criterion |
|---|---|---|---|
| Case evolution | Consistency in the filling in of the Case evolution variable | Filed filled in with valid codes (1 to 6), as per official standardization: 1=High with seroconversion; 2=High without seroconversion; 3=High source negative; 4=Dropout; 5=Death due to exposure; 6=Death due to other cause | Filed left blank, filled in with an invalid code or with “Unknown” code (code 9) |
