# Supplementary material for: Completeness, consistency and duplication of records of accidents involving exposure to biological material in dental settings: a descriptive study, Belo Horizonte, Brazil, 2006-2019
Source: Epidemiol Serv Saude. 2026 May 8;35:e20250762. doi: 10.1590/S2237-962220v35e20250762.en (PMC13686414; doi:10.1590/S2237-962220v35e20250762.en)
Supplement: Supplementary file 4 [file 2237-9622-ress-35-e2025762-Tabelas-Suplementares-PT.pdf]

Tabela suplementar 1. Variáveis analisadas e respectivas categorias em registros de acidentes com exposição a material biológico em ambientes odontológicos. Belo Horizonte, 2006-2019

Nome da variável	Classificação	Categorias/valores (códigos)[a]
**Variáveis de identificação da notificação e do evento:**		
Número da notificação	Obrigatória	Campo aberto (identificador do sistema)
Nome do agravo/doença	Obrigatória	Acidente de trabalho com exposição a material biológico (texto fixo)
Data da notificação	Obrigatória	Data (DD/MM/AAAA)
Sigla da unidade federada[b]	Obrigatória	Sigla e/ou código Instituto Brasileiro de Geografia e Estatística
Município da unidade notificadora[b]	Obrigatória	Nome do município e código Instituto Brasileiro de Geografia e Estatística
Cadastro nacional de estabelecimentos de saúde da unidade notificadora[b]	Obrigatória	Código específico
Data do acidente	Obrigatória	Data (DD/MM/AAAA)
**Variáveis demográficas e de residência:**		
Nome completo do paciente	Obrigatória	Campo aberto
Data de nascimento	Obrigatória	Data (DD/MM/AAAA)
Idade (se data de nascimento desconhecida)	Obrigatória	Número (anos)
Sexo do paciente	Obrigatória	Masculino (M); Feminino (F); Ignorado (I)
Gestante	Obrigatória	1-6; 9 (descrição conforme ficha Sinan 2019)
Unidade federada da residência do paciente[b]	Obrigatória	UF (sigla) e/ou código Instituto Brasileiro de Geografia e Estatística
Código do município de residência	Obrigatória	Código Instituto Brasileiro de Geografia e Estatística do município
País de residência (se estrangeiro)[b]	Obrigatória	Campo aberto (nome do país)
Identificação da ocupação	Obrigatória	Campo aberto (descrição); se disponível, código CBO-2002
Emissão de comunicação de acidente de trabalho (CAT)	Obrigatória	1=Sim; 2=Não; 3=Não se aplica; 9=Ignorado
Raça/cor da pele	Essencial	1=Branca; 2=Preta; 3=Amarela; 4=Parda; 5=Indígena; 9=Ignorado
Nível de escolaridade	Essencial	0-10 (descrição conforme ficha Sinan 2019)
Nome completo da mãe; nome do distrito da residência; nome do bairro; tipo de logradouro; número do logradouro; complemento; ponto de referência	Essencial	Campos abertos
Código de endereçamento postal (CEP)	Essencial	CEP (numérico)
Código de discagem direta à distância (DDD)	Essencial	DDD (numérico)
Telefone do paciente	Essencial	Número do telefone (numérico)
Zona de residência do paciente	Essencial	1=Urbana; 2=Rural; 3=Periurbana; 9=Ignorado
**Variáveis laborais:**		
Situação no mercado de trabalho	Essencial	01-12 (descrição conforme ficha Sinan 2019)
Tempo de trabalho na ocupação	Essencial	Número de anos (campo aberto)

Identificação da empresa contratante	Essencial	CNPJ/CPF (campo aberto)
Razão social da empresa contratante[b]	Essencial	Campo aberto
Classificação nacional de atividades econômicas[b]	Essencial	Código específico
Unidade federada da localização da empresa[b]	Essencial	Sigla e/ou código Instituto Brasileiro de Geografia e Estatística
Município do contratante[b]	Essencial	Nome do município e código Instituto Brasileiro de Geografia e Estatística
Distrito do município[b]/Bairro do município[b]/Endereço da empresa[b]/Número das instalações da empresa[b]/	Essencial	Campos abertos
Empresa terceirizada?	Essencial	1=Sim; 2=Não; 3=Não se aplica; 9=Ignorado
**Tipo de exposição[c]:**		
Percutânea/Mucosa/Pele íntegra/Pele íntegra/Outras	Essencial	Para cada subitem: 1=Sim; 2=Não; 9=Ignorado
**Caracterização do acidente:**		
Tipo de material orgânico	Essencial	1-8; 9 (descrição conforme ficha Sinan 2019)
Circunstância do acidente	Essencial	Código conforme ficha do Sinan (2019): 01-16, 98=Outros, 99=Ignorado. (descrições no instrumento oficial)
Agente causador do acidente	Essencial	1=Agulha com lúmen; 2=Agulha sem lúmen/maciça; 3=Intracath; 4=Vidros; 5=Lâmina/lanceta; 6=Outros; 9=Ignorado
**Uso de equipamento de proteção individual[c]:**		
Luvas/Avental/Óculos/Máscara/Proteção facial/Bota	Essencial	Para cada subitem: 1=Sim; 2=Não; 9=Ignorado
**Exames laboratoriais/Situação vacinal:**		
Situação vacinal contra hepatite B	Essencial	1=Vacinado; 2=Não vacinado; 9=Ignorado (esquema 3 doses)
**Resultados de exames (acidentado e fonte) (Data zero):**		
Painel sorológico (Anti-HIV, HBsAg, Anti-HBs, Anti-HCV)	Essencial	Para cada subitem: 1=Positivo; 2=Negativo; 3=Inconclusivo; 4=Não realizado; 9=Ignorado
Paciente-fonte conhecido	Essencial	1=Sim; 2=Não; 9=Ignorado
**Conduta no momento do acidente[c]:**		
Itens múltiplos (variáveis dicotômicas): quimioprofilaxia/ARV/esquemas/vacina/IGHB	Essencial	Para cada subitem: 1=Sim; 2=Não; 9=Ignorado
**Evolução do caso**	Essencial	1-6; 9 (descrição conforme ficha Sinan 2019)
**Óbito (se aplicável)**	Essencial	Capturado por 'Evolução do caso' (5 ou 6); 'Data do óbito' é campo de data (DD/MM/AAAA)

Nota: [a]Categorias e códigos extraídos da ficha de investigação "Acidente de trabalho com exposição a material biológico" do Sistema de Informação de Agravos de Notificação; [b]Campos sem codificação oficial indicados como "campo aberto" ou como "identificador externo", utilizando os seguintes sistemas: Instituto Brasileiro de Geografia e Estatística, Cadastro Nacional de Estabelecimentos de Saúde, Classificação Nacional de Atividades Econômicas e Classificação Brasileira de Ocupações; [c]Em grupos compostos por múltiplos itens, cada subitem é registrado como variável dicotômica independente. Brasil. Ministério da Saúde. Secretaria de Vigilância em Saúde. Ficha de investigação: Acidente de trabalho com exposição a material biológico [Internet]. Brasília: Ministério da Saúde; 2019 Jun 21 [cited 2025 Sep 25]. Available from: https://portalsinan.saude.gov.br/images/DRT/DRT_Acidente_Trabalho_Biologico.pdf.

Tabela suplementar 2. Critérios de consistência entre variáveis em registros de acidentes com exposição a material biológico em ambientes odontológicos. Belo Horizonte, 2006-2019

Par de variáveis	Descrição da relação esperada	Critério de consistência	Critério de inconsistência
Sexo do paciente × Gravidez	Consistência entre sexo e preenchimento da variável Gravidez	Registros com sexo masculino e variável Gravidez preenchida como “Não aplicável” (código 6)	Registros com sexo feminino e variável Gravidez preenchida com código incompatível (“Não aplicável”, código 6)
Tipo de exposição × Circunstância do acidente	Consistência entre tipo de exposição e circunstância do acidente informada	Para cada tipo de exposição registrada (Mucosa, Pele íntegra, Pele não íntegra ou Percutânea), circunstância do acidente compatível conforme os códigos padronizados do sistema	Tipo de exposição incompatível com a circunstância do acidente, ou registros classificados como “Ignorado” (códigos 9 ou 99) de forma inconsistente
Paciente-fonte conhecido × Testes laboratoriais do paciente-fonte	Consistência entre identificação de paciente-fonte e realização de exames laboratoriais	Para registros com paciente-fonte identificado como “Sim” (código 1), pelo menos um teste laboratorial informado com valor válido	Para registros com paciente-fonte identificado como “Sim” (código 1), todos os testes laboratoriais em branco, marcados como “Não realizado” (código 4) ou “Ignorado” (código 9)
Idade do paciente (anos) × Tempo de ocupação (anos)	Consistência entre idade do paciente e tempo de ocupação registrado	Tempo de ocupação informado igual ou inferior à idade atual do paciente e valor não negativo	Tempo de ocupação maior que a idade atual do paciente ou valor negativo
Terceirização × Nome da empresa contratante	Consistência entre informação sobre terceirização e preenchimento do campo Nome da empresa contratante	Para registros com Terceirização informada como “Sim” (código 1), campo Nome da empresa contratante preenchido; para Terceirização=“Ignorado” (código 9), campo Nome da empresa contratante em branco	Para registros com Terceirização=“Sim” (código 1), campo Nome da empresa contratante em branco; ou para Terceirização=“Ignorado” (código 9), campo Nome da empresa contratante preenchido

Par de variáveis	Descrição da relação esperada	Critério de consistência	Critério de inconsistência
Evolução do caso	Consistência no preenchimento da variável Evolução do caso	Campo preenchido com códigos válidos (1 a 6), conforme padronização oficial: 1=Alta com conversão sorológica; 2=Alta sem conversão; 3=Alta fonte negativa; 4=Abandono; 5=Óbito por exposição; 6=Óbito por outra causa	Campo em branco, preenchido com código inválido ou com código “Ignorado” (código 9)
