# Supplementary material for: Completeness, consistency and duplication of records of accidents involving exposure to biological material in dental settings: a descriptive study, Belo Horizonte, Brazil, 2006-2019
Source: Epidemiol Serv Saude. 2026 May 8;35:e20250762. doi: 10.1590/S2237-962220v35e20250762.en (PMC13686414; doi:10.1590/S2237-962220v35e20250762.en)
Supplement: Translation

# Translation: Completude, consistência e duplicidade dos registros de acidentes com exposição a material biológico em ambientes odontológicos: estudo descritivo, Belo Horizonte, 2006-2019

**Authors:** Ana Carolina Marques Medeiros, Victor Santos Batista, Luis Otávio Pereira da Silva, Juliana Vaz de Melo Mambrini, Rafaela Silveira Pinto, Mauro Henrique Nogueira Guimarães Abreu

**Affiliations:** 1 Universidade Federal de Minas Gerais, Belo Horizonte, MG, Brasil Universidade Federal de Minas Gerais Belo Horizonte MG Brasil; 2 Fundação Oswaldo Cruz Instituto René Rachou, Belo Horizonte, MG, Brasil Fundação Oswaldo Cruz Instituto René Rachou Belo Horizonte MG Brasil

**Keywords:** Exposição Ocupacional, Odontologia, Acidentes de Trabalho, Qualidade dos Dados, Estudos Descritivos

## Abstract

**Objetivo:**

Avaliar a duplicidade, a completude e a consistência interna dos registros de acidentes com exposição a material biológico em ambientes odontológicos em Belo Horizonte de 2006 a 2019.

**Método:**

Estudo descritivo com dados do Sistema de Informação de Agravos de Notificação, com inclusão de variáveis de identificação, dados sociodemográficos e laborais, circunstâncias do acidente, uso de equipamentos de proteção, exames laboratoriais, conduta e evolução. A duplicidade foi identificada por combinações determinísticas e probabilísticas. A completude foi classificada como excelente (≥95%), boa (90%-94,9%), regular (70%-89,9%), ruim (50%69,9%) e muito ruim (<50%). A consistência foi avaliada em pares de variáveis e classificada como excelente (≥90%), regular (70%-89%) e baixa (<70%).

**Resultados:**

Foram investigados 1.054 registros, sem duplicidades identificadas. Entre as 17 variáveis obrigatórias analisadas, 15 apresentaram completude excelente; "gestante" (80,4%) e "emissão da comunicação de acidente de trabalho" (75,5%) foram classificadas como regulares. Entre as variáveis essenciais, os campos relacionados a equipamentos de proteção individual alcançaram completude regular (78,0%-89,9%), enquanto o campo exames laboratoriais do acidentado e do paciente-fonte, bem como "conduta no momento do acidente" e "evolução do caso", exibiram completude muito ruim (<50%). Na consistência, os pares "paciente-fonte conhecido/resultados dos testes do paciente-fonte" e "tipo de exposição/agente causador" foram classificados como regulares, e "evolução do caso" demonstrou baixa consistência (55,1%).

**Conclusão:**

Embora o sistema registre adequadamente a ocorrência dos acidentes, fragilidades em variáveis clínicas e laborais reduzem sua utilidade para a vigilância, já que restringem a avaliação do risco de infecção e o seguimento clínico dos trabalhadores expostos.

**Aspectos éticos**

A presente pesquisa respeitou os princípios éticos, obtendo os seguintes dados de aprovação:

Comitê de ética em pesquisa: Universidade Federal de Minas Gerais

Números dos pareceres: 6.097.439

Datas de aprovação: 2/6/2023

Comitê de ética em pesquisa: Secretaria Municipal de Saúde de Belo Horizonte

Números dos pareceres: 6.161.565

Datas de aprovação: 4/7/2023

Certificado de apresentação de apreciação ética

68702123.9.0000.5149

68702123.9.3001.5140

Registro do consentimento livre e esclarecido: Dispensado.

## Introdução 

A exposição a material biológico constitui um problema global entre profissionais da saúde, com prevalência acumulada ao longo da carreira de 56,6%^1^. Esses acidentes implicam risco de soroconversão ao HIV, vírus da hepatite B e vírus da hepatite C, além de custos assistenciais, afastamentos do trabalho e impactos psicológicos^2^. No campo da odontologia, esse risco é particularmente elevado, dado o uso frequente de instrumentos perfurocortantes e a realização de procedimentos invasivos^3^. Revisões sistemáticas indicam prevalências globais de acidentes percutâneos de 44% entre estudantes de odontologia^4^ e proporção semelhante entre auxiliares, especialmente em atividades como limpeza de instrumentais e manuseio de seringas de anestesia, consideradas de maior risco^5^. Em comparação com diferentes categorias de profissionais da saúde, cirurgiões-dentistas apresentaram a maior prevalência, um total de 59% das notificações.^3^

No Brasil, entre 2006 e 2022, foram notificados 36.171 (4,5%) acidentes de trabalho decorrentes de procedimentos odontológicos^6^, o que reforçou a magnitude do problema no país. Em Belo Horizonte, Minas Gerais, no período de 2019 a 2024, a plataforma de tabulação do Departamento de Informática do Sistema Único de Saúde identificou 8.607 acidentes, dos quais 366 (4,3%) ocorreram durante procedimentos odontológicos^7^. Esses dados evidenciam a relevância da vigilância contínua desse agravo no contexto da saúde do trabalhador.

Diante da magnitude dos acidentes notificados no país e no município, torna-se essencial o monitoramento sistemático dos casos. Em âmbito nacional, o Sistema de Informação de Agravos de Notificação (Sinan) constitui a principal ferramenta para acompanhamento dos agravos e orientação de ações de vigilância^8^^,^^9^. A qualidade das informações registradas pode ser avaliada a partir de três atributos fundamentais: completude, consistência interna e duplicidade^10^^,^^11^. Entre 2010 e 2015, análises das notificações de acidentes com exposição a material biológico revelaram variações na classificação da completude das variáveis essenciais, que oscilaram entre excelente e muito ruim, sem que os atributos de consistência ou duplicidade fossem considerados^12^. 

Embora a qualidade dos sistemas de informação em saúde venha ganhando destaque nas discussões sobre vigilância, os acidentes com exposição a material biológico em ambientes odontológicos demandam análises específicas que considerem o desempenho dos registros frente a atributos essenciais de qualidade. Avaliar esses componentes do sistema de notificação é fundamental para qualificar o uso dos dados na formulação de ações e de políticas de saúde. Este estudo objetivou, nesse contexto, avaliar a duplicidade, a completude e a consistência interna dos registros de acidentes com exposição a material biológico em ambientes odontológicos em Belo Horizonte no período de 2006 a 2019.

## Métodos

Métodos Delineamento

Trata-se de estudo descritivo. 

Este estudo foi reportado conforme a diretriz *REporting of studies Conducted using Observational Routinely-collected health Data*^13^.

Contexto

Foram utilizados dados através do Sinan sobre acidentes com exposição a materiais biológicos em ambientes odontológicos de Belo Horizonte no período de 2006 a 2019. Esse intervalo corresponde à disponibilidade do banco municipal informatizado e antecede as distorções provocadas pela pandemia de covid-19^14^. O acesso ao banco de dados ocorreu em 1º de agosto de 2023. 

O banco foi fornecido pela Secretaria Municipal de Saúde de Belo Horizonte, sem exclusão prévia de duplicidades, e a seleção final foi validada pela Coordenação de Saúde do Trabalhador.

Participantes

Os participantes corresponderam às notificações de acidentes com exposição a material biológico registradas em ambientes odontológicos no município de Belo Horizonte. Foram incluídos registros que apresentaram ao menos uma das seguintes características: a ocupação, segundo a Classificação Brasileira de Ocupação, de cirurgião-dentista (código 2.232), técnico de saúde bucal, auxiliar de saúde bucal, atendente ou auxiliar odontológico, (código 3.224); a Classificação Nacional de Atividades Econômicas na área odontológica (códigos 8630-5/04, 8650-0/03 e outros identificados por evidências complementares); a circunstância do acidente (código 13); ou menções nos campos de observação por filtros textuais ("odont*" ou "dent*"). Notificações que não se enquadravam em pelo menos uma dessas quatro características anteriores foram excluídas. 

Variáveis

As variáveis foram classificadas em obrigatórias (campos cujo preenchimento é indispensável para a validação da notificação) e essenciais (informações relevantes para análise epidemiológica, mas não impeditivas da digitação), conforme definições do sistema^8^^,^^9^. A classificação e os códigos do sistema estão descritos na Tabela suplementar 1. As variáveis seguiram as definições do manual técnico Acidente com Exposição a Material Biológico^9^, elaborado pela Secretaria da Saúde do Estado do Ceará, adotado por tratar de forma específica esse agravo. As definições foram utilizadas em consonância com manuais do Ministério da Saúde^8^^,^^15^, que apresentam orientações conceituais e metodológicas voltadas à vigilância de agravos e à avaliação da qualidade das informações em sistemas de saúde. 

Fonte de dados e mensuração

A qualidade dos dados foi analisada levando-se em consideração três atributos: duplicidade^10^^,^^11^, completude^10^^,^^11^e consistência^10^^,^^11^, conforme operacionalizado em estudos nacionais^10^^,^^11^. A duplicidade foi definida como registros do mesmo acidente notificados mais de uma vez para o mesmo paciente em curto intervalo^8^. A verificação ocorreu em duas etapas^8^: determinística, pelo cruzamento direto de nome, data de nascimento, sexo e data do acidente; e probabilística, com uso de algoritmos Soundex e de fonética para identificar registros semelhantes, apesar de variações ortográficas.

A completude foi avaliada pela proporção de preenchimento válido em cada variável, com a compreensão de que incompletos são campos em branco e campos codificados como "ignorado"^8^. Foram aplicados os pontos de corte adotados pelo manual do Ministério da Saúde^8^, que classifica cada variável avaliada pelo percentual de preenchimento completo: ≥95% (excelente); 90%-94,9% (bom); 70%-89,9% (regular); 50%-69,9% (ruim); <50% (muito ruim). Para a variável gestante, a categoria "não se aplica", atribuída a registros masculinos, foi considerada como preenchimento válido^8^.

A consistência interna foi definida como a coerência entre variáveis que devem apresentar relação lógica^8^ com base em fundamentos técnico-epidemiológicos do manual de exposição a material biológico do Ministério da Saúde^15^ e nos manuais de qualidade do sistema^8^^,^^9^. Foram testadas as combinações: sexo e gravidez; tipo de exposição e circunstância do acidente; tipo de exposição e agente causador; paciente-fonte conhecido e resultados laboratoriais; idade e tempo de ocupação; terceirização e nome da empresa contratante; evolução do caso. Os critérios operacionais e exemplos de incompatibilidades estão descritos na Tabela suplementar 2. Quando a consistência das combinações apresentou-se ≥90%, foi classificada como excelente; quando ficou entre 70%-89%, foi considerada regular; e quando foi <70%, foi classificada como baixa^16^.

Controle de viés 

Adotou-se uma janela temporal de até sete dias entre notificações de um mesmo indivíduo como critério complementar para detecção de repetições decorrentes de erros de digitação, sem confundir eventos distintos. Esse intervalo foi definido sob o entendimento de que o manual próprio do sistema exemplifica duplicidades em agravos agudos quando notificações ocorrem na mesma semana epidemiológica, unidade temporal oficialmente equivalente a sete dias consecutivos^17^. Como acidentes com material biológico são eventos agudos e de notificação imediata^17^, a adoção desse intervalo é coerente com a natureza e o ciclo operacional do agravo. Registros fora desse período também foram verificados pelas etapas determinística e probabilística, o que garantiu a identificação de duplicidades não temporais.

 Tamanho do estudo

A população do estudo foi composta por todas as notificações de acidentes com exposição a material biológico em ambientes odontológicos em Belo Horizonte.

Métodos Estatísticos

Os dados foram disponibilizados em formato Excel (Microsoft Corp., Redmond, WA, USA) e processados no Python (versão 3.13.1; Python Software Foundation) com uso das bibliotecas Pandas (2.2.2) e NumPy (1.26.4). As etapas incluíram padronização de categorias e códigos de valores ignorados, uniformização de textos, remoção de espaços em branco, conversão de datas para formato adequado, cálculo de idade no momento do acidente e de tempo de ocupação em anos inteiros. Também foram criadas variáveis derivadas, como a chave composta para detecção de duplicidades. Quando a idade estava ausente, foi recalculada a partir da data de nascimento e da data do acidente. Valores impossíveis, como tempo de ocupação superior à idade, foram recodificados como inválidos. As etapas de validação, padronização e depuração do banco consolidado estão sintetizadas na Figura suplementar 1.

Os resultados foram expressos em números absolutos e percentuais para duplicidade, completude e consistência. Scripts e documentação do fluxo metodológico constam no material suplementar.

## Resultados 

Do total de 12.717 registros de acidentes com exposição a material biológico, 1.054 atenderam aos critérios de inclusão do estudo e compuseram a análise (Figura suplementar 2). Não foram observadas duplicidades, e dois registros adicionais corresponderam a acidentes distintos em um mesmo trabalhador.

A Tabela 1 apresentou a completude das variáveis obrigatórias e essenciais. Entre as variáveis obrigatórias, verificou-se que 15 dos 17 campos apresentaram completude classificada como excelente, enquanto "gestante" (80,4%) e "emissão da comunicação de acidente de trabalho" (75,5%) foram classificadas como regulares. Entre as variáveis essenciais, campos relacionados ao endereço (complemento do logradouro, ponto de referência e código de endereçamento postal) exibiram completude com variação entre ruim e muito ruim, enquanto "código de discagem direta à distância" foi classificado como regular. Variáveis de contato, como telefone e zona de residência, foram classificadas como regulares. Em relação as 12 variáveis laborais, houve predomínio das classificações ruim (41,6%) e regular (33,3%).

A Tabela 2 sintetizou as variáveis essenciais de exposição: caracterização do acidente, uso de equipamentos de proteção individual (EPI) e exames laboratoriais. Observou-se boa proporção de registros para acidentes percutâneos (90,8%), proporção ruim de tipo de exposição mucosa (52,4%) e muito ruim de pele não íntegra (49,7%). Variáveis relacionadas ao uso de EPI, inclusive luvas, avental, óculos e máscara, bem como "paciente-fonte conhecido", apresentaram completude regular (78,0%-89,9%). As demais variáveis clínicas exibiram as menores proporções de preenchimento e todas foram classificadas como de completude muito ruim (<50%). 

A Tabela 3 apresentou os resultados da avaliação de consistência. A maior parte das combinações foi classificada como excelente, enquanto "paciente-fonte conhecido e resultados laboratoriais" (13,1%) e "tipo de exposição e agente causador" (13,5%) apresentaram consistência regular. A variável "evolução do caso" exibiu consistência baixa (55,1%).

**Tabela 1:** Completude das variáveis de identificação, demográficas, de residência e laborais em registros de acidentes com exposição a material biológico em ambientes odontológicos. Belo Horizonte, 2006-2019 (n=1.054)

Variável	n (%)	Classificação^a^
Obrigatórias		
Variáveis de identificação da notificação e do evento		
Número da notificação	1.054 (100,0)	Excelente
Nome do agravo/doença	1.054 (100,0)	Excelente
Data da notificação	1.054 (100,0)	Excelente
Sigla da unidade federada	1.054 (100,0)	Excelente
Município da unidade notificadora	1.054 (100,0)	Excelente
Cadastro nacional de estabelecimentos de Saúde da unidade notificadora	1.054 (100,0)	Excelente
Data do acidente	1.054 (100,0)	Excelente
Variáveis demográficas e de residência		
Nome completo do paciente	1.054 (100,0)	Excelente
Data de nascimento	1.054 (100,0)	Excelente
Idade (anos, se data de nascimento desconhecida)	1.054 (100,0)	Excelente
Sexo do paciente	1.054 (100,0)	Excelente
Gestante	847 (80,4)	Regular
Unidade federada da residência do paciente	1.054 (100,0)	Excelente
Código do município de residência	1.054 (100,0)	Excelente
País de residência (se estrangeiro)	1.054 (100,0)	Excelente
Identificação da ocupação	1.054 (100,0)	Excelente
Emissão da comunicação de acidente de trabalho	796 (75,5)	Regular
Essenciais		
Raça/cor da pele	909 (86,2)	Regular
Nível de escolaridade	1.040 (98,7)	Excelente
Nome completo da mãe	1.054 (100,0)	Excelente
Nome do distrito da residência	854 (81,0)	Regular
Nome do bairro	1.047 (99,2)	Excelente
Tipo de logradouro da residência	1.046 (99,1)	Excelente
Número do logradouro	1.039 (98,6)	Excelente
Complemento do logradouro	556 (52,8)	Ruim
Ponto de referência da residência	274 (26,0)	Muito ruim
Código de endereçamento postal	649 (61,6)	Ruim
Código de discagem direta à distância	868 (82,3)	Regular
Telefone do paciente	866 (82,2)	Regular
Zona de residência do paciente	881 (83,5)	Regular
Variáveis laborais		
Situação no mercado de trabalho	985 (93,5)	Bom
Tempo de trabalho na ocupação (anos)	1.039 (98,6)	Excelente
Identificação da empresa contratante	628 (59,6)	Ruim
Razão social da empresa contratante	1.054 (100,0)	Excelente
Classificação nacional de atividades econômicas	757 (71,8)	Regular
Unidade federada da localização da empresa	844 (80,1)	Regular
Município do contratante	808 (76,7)	Regular
Distrito do município	689 (65,4)	Ruim
Bairro do município	691 (65,6)	Ruim
Endereço da empresa	694 (65,8)	Ruim
Número das instalações da empresa	689 (65,4)	Ruim
Empresa terceirizada	893 (84,7)	Regular

a Classificação da completude: Excelente ≥95%; Bom 90%-94,9%; Regular 70%-89,9%; Ruim 50%-69,9%; Muito ruim <50%.

**Tabela 2:** Completude das variáveis de exposição, caracterização do acidente e desfechos clínicos em acidentes com material biológico em ambientes odontológicos. Belo Horizonte, 2006-2019 (n=1.054)

Variável	n (%)	Classificação^a^
Essenciais		
Tipo de exposição		
Percutânea	957 (90,8)	Bom
Mucosa	552 (52,4)	Ruim
Pele íntegra	640 (60,7)	Ruim
Pele não íntegra	524 (49,7)	Muito ruim
Outras	347 (32,9)	Muito ruim
Caracterização do acidente		
Tipo de material orgânico	954 (90,5)	Bom
Circunstância do acidente	1.038 (98,5)	Excelente
Agente causador do acidente	997 (94,6)	Bom
Uso de equipamento de proteção individual		
Luvas	947 (89,9)	Regular
Avental	840 (79,7)	Regular
Óculos	822 (78,0)	Regular
Máscara	836 (79,3)	Regular
Proteção facial	719 (68,2)	Ruim
Bota	695 (65,9)	Ruim
Exames laboratoriais/situação vacinal		
Situação vacinal contra hepatite B	873 (82,8)	Regular
Resultados de exames do acidentado (data zero)		
Teste para detecção de anticorpos contra o vírus da imunodeficiência humana	517 (49,0)	Muito ruim
Antígeno de superfície do vírus da hepatite B	484 (45,9)	Muito ruim
Teste para detecção de anticorpos contra o antígeno de superfície do vírus da hepatite B	437 (41,4)	Muito ruim
Teste para detecção de anticorpos contra o vírus da hepatite C	483 (45,8)	Muito ruim
Paciente-fonte conhecido	922 (87,4)	Regular
Resultados de exames do paciente-fonte		
Antígeno de superfície do vírus da hepatite B	394 (37,4)	Muito ruim
Teste para detecção de anticorpos contra o vírus da imunodeficiência humana	454 (43,0)	Muito ruim
Teste para detecção de anticorpos contra o antígeno do núcleo (core) do vírus da hepatite B	326 (30,9)	Muito ruim
Teste para detecção de anticorpos contra o vírus da hepatite C	368 (34,9)	Muito ruim
Conduta no momento do acidente		
Sem indicação de quimioprofilaxia	449 (42,6)	Muito ruim
Recusou quimioprofilaxia indicada	103 (9,8)	Muito ruim
Zidovudina e lamivudina	199 (18,9)	Muito ruim
Zidovudina, lamivudina e indinavir	133 (12,6)	Muito ruim
Zidovudina, lamivudina e nelfinavir	107 (10,1)	Muito ruim
Imunoglobulina humana contra hepatite B	107 (10,1)	Muito ruim
Vacina contra hepatite B	108 (10,2)	Muito ruim
Outro esquema de antirretrovirais	335 (31,8)	Muito ruim
Especificação do outro esquema	155 (14,7)	Muito ruim
Evolução do caso	473 (44,9)	Muito ruim

a Classificação da completude: Excelente ≥95%; Bom 90%-94,9%; Regular 70%-89,9%; Ruim 50%-69,9%; Muito ruim <50%.

**Tabela 3:** Inconsistências entre variáveis em registros de acidentes com exposição a material biológico em ambientes odontológicos. Belo Horizonte, 2006-2019 (n=1.054)

Critério de consistência	n^a^ (%^a,b^)	Classificação^c^
Sexo e gravidez	59 (5,6)	Excelente
Idade (anos) e tempo na ocupação (anos)	94 (9,0)	Excelente
Paciente-fonte conhecido e resultados dos testes do paciente-fonte	138 (13,1)	Regular
Tipo de exposição e agente causador	147 (13,5)	Regular
Tipo de exposição e circunstância do acidente	38 (3,6)	Excelente
Terceirização e nome da empresa contratante	8 (0,8)	Excelente
Evolução do caso	581 (55,1)	Baixa

a Refere-se aos registros inconsistentes; ^b^Percentuais calculados com base nos registros com ambas as variáveis preenchidas; ^c^Classificação da consistência: Excelente ≥90% de registros consistentes; Regular 70%-89%; Baixa consistência <70%.

## Discussão 

A avaliação dos registros de acidentes com exposição a material biológico em ambientes odontológicos em Belo Horizonte no período de 2006 a 2019 evidenciou ausência de duplicidades e completude excelente na maioria das variáveis obrigatórias. Para as variáveis essenciais, observou-se uma variabilidade no Sinan, de muito ruim a excelente. Importantes variáveis para o evento, como caracterização do acidente e desfechos clínicos de acidentes com material biológico em ambientes odontológicos, tiveram, frequentemente, desempenho muito ruim e ruim. A consistência interna foi classificada como regular entre "paciente-fonte conhecido e resultados laboratoriais" e "tipo de exposição e agente causador"; e houve registro de baixa coerência para "evolução do caso". Esses achados são preocupantes para o seguimento e a vigilância epidemiológica dos profissionais de saúde bucal acidentados.

Ressalta-se que os resultados deste estudo devem ser interpretados com a consideração das limitações inerentes ao delineamento descritivo e ao uso de dados secundários, sujeitos a viés de classificação, incompletude e subnotificação, especialmente em acidentes recorrentes ou não formalmente comunicados. Apesar dessas restrições, os achados ofereceram subsídios consistentes para aprimorar o processo de notificação, fortalecer a vigilância e orientar estratégias de prevenção. No tocante à duplicidade, a ausência de registros repetidos sugeriu adequado controle do fluxo de notificações e consolidação efetiva dos dados, componentes essenciais para sistemas de vigilância^18^. Achados semelhantes foram descritos na hanseníase em João Pessoa, Paraíba, entre 2001 e 2019, em que foi identificado 0,3% de duplicidades^10^, e em notificações de violência sexual contra crianças e adolescentes em Santa Catarina, de 2009 a 2019, nas quais também não foram identificados registros duplicados^18^, o que reforçou a efetividade de rotinas de depuração. Esses resultados convergem para a importância do aperfeiçoamento contínuo de rotinas de duplicação e da vigilância ativa. 

A identificação de dois acidentes distintos no mesmo trabalhador reafirmou a necessidade de distinguir repetições indevidas de recorrências reais, que indicam risco ocupacional persistente. Essa distinção depende de regras de vinculação e verificação temporal aplicadas de forma criteriosa^19^. Janelas temporais curtas podem não captar todas as repetições, o que evidenciou a importância de auditorias e realimentação periódica do sistema^8^^,^^9^^,^^20^. 

A completude das variáveis obrigatórias mostrou-se majoritariamente excelente, resultado semelhante ao observado nacionalmente entre 2010-2015 para variáveis de identificação em acidentes com exposição a material biológico^12^. Esses resultados indicaram que a obrigatoriedade técnica favorece o preenchimento, embora não assegure qualidade ou precisão dos registros, que dependem do entendimento e do comprometimento dos notificadores^8^^,^^12^^,^^21^. As variáveis "gestante" e "emissão da comunicação de acidente de trabalho" apresentaram completude regular, o que reforçou que a presença formal do campo não assegura o seu registro. Em nível nacional, de 2010 a 2015, a completude do campo "comunicação de acidente de trabalho" foi <70%^12^, e em hospital universitário de Pernambuco, de 2009 a 2019, 98% das fichas não continham essa informação, o que inviabilizou a análise de aspectos legais e trabalhistas^22^. 

Esses resultados refletiram a falta de integração entre vigilância e setor trabalhista, o que dificultou a rastreabilidade dos acidentes^22^. A variável "gestante" também se mostrou crítica, pois o protocolo nacional prevê acompanhamento específico e ajustes profiláticos^15^. Em ambientes odontológicos, onde mulheres predominam entre os expostos e acidentes percutâneos são frequentes durante a limpeza de instrumentais e a manipulação de seringas anestésicas , a ausência do dado restringiu a vigilância de riscos específicos^5^^,^^23^.

Entre as variáveis essenciais, exames laboratoriais do acidentado e da fonte e conduta e evolução apresentaram completude ruim e muito ruim, padrão semelhante ao nacional^12^. Essas deficiências de preenchimento sugeriram dificuldades de articulação entre vigilância e assistência, uma vez que o acompanhamento clínico nem sempre é registrado como parte do processo de notificação^8^^,^^22^. 

O tipo de exposição, variável determinante do risco de infecção^15^^,^^20^, apresentou boa completude para acidentes percutâneos, mas desempenho ruim ou muito ruim para exposições mucosas e cutâneas. Esse padrão repetiu achados nacionais^12^ e revisões internacionais em odontologia^3^^,^^23^. Por envolver maior risco de transmissão de vírus HIV, HBV e HCV, esse tipo de acidente tende a ser mais prontamente notificado, o que pode refletir maior percepção de risco entre os profissionais^12^^,^^22^^,^^23^. 

A ausência de elevado percentual de resultados laboratoriais e de evolução, como os encontrados no estudo, também podem comprometer a vigilância e inviabilizar o acompanhamento da soroconversão, essencial para caracterizar o risco real de infecção^12^^,^^15^^,^^22^. Essa lacuna pode decorrer de falhas de registro, quando exames e condutas são realizados, mas não informados, ou da ausência efetiva de testagem e seguimento. No primeiro caso, há perda de confiabilidade das estatísticas^8^^,^^12^^,^^21^; no segundo, há falha assistencial, em desacordo com protocolos que preveem testagem da fonte e do acidentado e acompanhamento por até seis meses^15^^,^^20^. Revisões internacionais associaram baixa completude à perda de utilidade secundária dos dados, especialmente em campos clínicos^24^, e reforçaram a necessidade de rotinas de controle e interfaces mais funcionais^24^^,^^25^.

A variável "conduta", que registra as medidas adotadas após o acidente, é essencial para avaliar o manejo pós-exposição^9^^,^^15^. Revisão recente relatou que cerca de um terço das fontes não é testado para vírus HIV, HBV ou HCV, geralmente por recusa ou falta de consentimento^26^. Estudos também apontaram subnotificação entre estudantes e auxiliares, o que dificulta avaliações pós-exposição^1^^,^^23^. Esse padrão sugere falhas persistentes na cultura de biossegurança, mesmo em cenários com diretrizes consolidadas^15^^,^^21^. 

A avaliação da "evolução do caso" também é crítica, já que é a responsável por indicar se houve acompanhamento ou abandono e elevada proporção de registros em branco. Isso sugere falhas de registro ou ausência de seguimento, em desacordo com protocolos nacionais e internacionais^9^^,^^15^^,^^21^, o que compromete a avaliação da efetividade da profilaxia indicada^21^^,^^22^.

Já para as variáveis laborais, a maioria variou a completude de regular a ruim, o que dificulta a identificação de ambientes de maior risco e limita o planejamento de medidas preventivas^27^. Essa limitação é relevante, porque condições de trabalho e adesão às normas influenciam a ocorrência e o registro das exposições^1^. A falta de informação sobre equipamentos de proteção individual também restringe análises de adesão às barreiras de proteção^15^^,^^23^. Estudo internacional demonstrou que variações na completude decorreram de falta de padronização, lacunas conceituais e capacitação insuficiente dos profissionais notificadores^28^.

No que se refere à consistência, a evolução do caso foi classificada como baixa, o que pode comprometer sua função de síntese dos desfechos e a utilidade do sistema para avaliar a efetividade das medidas profiláticas^15^^,^^20^. A consistência regular dos pares "paciente-fonte conhecido e resultados laboratoriais" e "tipo de exposição e agente causador" também pode afetar a avaliação do risco biológico e a definição de condutas pós-exposição^15^^,^^20^, além de dificultar o direcionamento das práticas preventivas^8^^,^^9^. A consistência é considerada uma das dimensões mais influentes da qualidade dos dados, pois afeta completude, acurácia, atualidade e acessibilidade^24^. Inconsistências na captura e ausência de padronização comprometem a coerência entre campos, como observado nos pares avaliados^24^. 

Compreender o cenário de fragilidades na completude e na consistência interna de variáveis essenciais em ambientes odontológicos é fundamental para identificar limitações do sistema e orientar estratégias mais efetivas de vigilância^26^. As fragilidades observadas indicaram desalinhamento entre normativas e práticas cotidianas, o que reforçou a necessidade de qualificação contínua do registro^8^^,^^21^. A vigilância pode ser fortalecida pela ampliação das ações ativas, o que assegura seguimento adequado e monitoramento dos desfechos^22^. Experiência internacional mostrou que a melhoria da qualidade depende de intervenções em todas as etapas do processo, como automatização das verificações, padronização de campos e capacitação periódica^28^. 

Sugere-se capacitação contínua das equipes, simplificação dos formulários e automação de rotinas de checagem, o que amplia a utilidade das informações para prevenção de acidentes, especialmente em cenários de maior risco. Avaliações periódicas do sistema permanecem indispensáveis para monitorar avanços e orientar ajustes contínuos^8^^,^^21^.

Em Belo Horizonte, Minas Gerais, os registros do Sinan demonstraram registrar adequadamente a ocorrência dos acidentes com exposição a material biológico em ambientes odontológicos, o que foi evidenciado pela ausência de duplicidades e pela identificação de recorrências reais. Mas persistiram fragilidades em variáveis clínicas e laborais, que reduzem a aplicabilidade do sistema para a vigilância em saúde. O preenchimento insuficiente e a inconsistência de campos como "exames laboratoriais", "conduta" e "evolução do caso" indicaram limitações estruturais no processo de notificação, o que compromete a avaliação do risco real de infecção e o seguimento clínico dos trabalhadores expostos. 

## Data Availability

Os dados analisados são administrativos e sigilosos, provenientes do Sistema de Informação de Agravos de Notificação sob a gestão da Secretaria Municipal de Saúde de Belo Horizonte. O acesso direto depende de autorização da autoridade competente. Os procedimentos de análise, com inclusão de arquivos gerados nas etapas de verificação e processamento, estão descritos neste manuscrito e no material suplementar. Solicitações de acesso poderão ser avaliadas pelos autores, conforme critérios legais de sigilo e apreciação editorial.

## SUPPLEMENTARY MATERIAL

Tabelas Suplementares

Figuras Suplementares
